# Supplementary material for: Historical deforestation drives strong rainfall decline across the southern Amazon basin
Source: Nat Commun. 2026 Jan 13;17:1642. doi: 10.1038/s41467-026-68361-z (PMC12905307; doi:10.1038/s41467-026-68361-z)
Supplement: Supplementary file 1 — Supplementary Information [file 41467_2026_68361_MOESM1_ESM.pdf]

Supplementary Information for

Historical deforestation drives strong rainfall decline across the southern Amazon basin

Jiangpeng Cui[1,*], Shilong Piao[2,*], Chris Huntingford[3], Tao Wang[1], Dominick V. Spracklen[4]

[1] State Key Laboratory of Tibetan Plateau Earth System, Environment and Resources (TPESER), Institute of Tibetan Plateau Research, Chinese Academy of Sciences, Beijing 100101, China.

[2] Institute of Carbon Neutrality, Sino-French Institute for Earth System Science, College of Urban and Environmental Sciences, Peking University, Beijing 100871, China.

[3] U.K. Centre for Ecology and Hydrology, Wallingford, Oxford shire, OX10 8BB, UK.

[4] School of Earth and Environment, University of Leeds, Leeds, UK

[*]Corresponding authors. Emails: cuijp@itpcas.ac.cn (J. Cui), slpiao@pku.edu.cn (S. Piao)

Contents of this file

Supplementary Figures 1 to 13

Supplementary Tables 1 to 3

Supplementary Fig. 1. Precipitation fraction that contributed from terrestrial recycled moisture. **a**, Spatial pattern of precipitation fraction that contributed from terrestrial recycled moisture (terrestrial precipitation recycling ratio) over the Amazon basin. The horizontal black line, at the latitude of 7.5ºS, indicates the division between the northern and southern Amazon basins, while the outer black curve defines the full spatial extent of the Amazon basin. Gray lines represent the isopleths of major terrestrial recycled precipitation fraction. **b**, Time series of terrestrial precipitation recycling ratio averaged over the southern Amazon basin. The black line is a fitted linear regression, and the shaded red areas represent the 95% confidence intervals of the regressions.

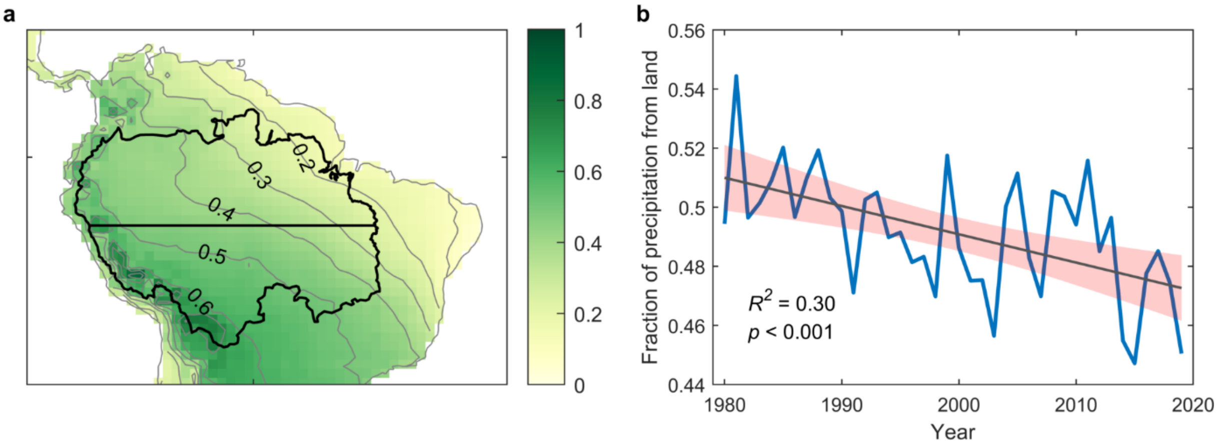

Supplementary Fig. 2. Trends in moisture sources of Amazon precipitation based on GPCC. a, Oceanic precipitation trend (P_oceanic). **b,** Terrestrial recycled precipitation trend (P_recycled). Stippling indicates regions where the trend is statistically significant ($p < 0.05$). **c,** Oceanic and terrestrial recycled precipitation trends averaged over the whole, northern and southern Amazon basins. Error bars represent the standard errors of the trends. Asterisks indicate that the trend is significant ($p < 0.05$). All trends are calculated for the period 1980-2019.

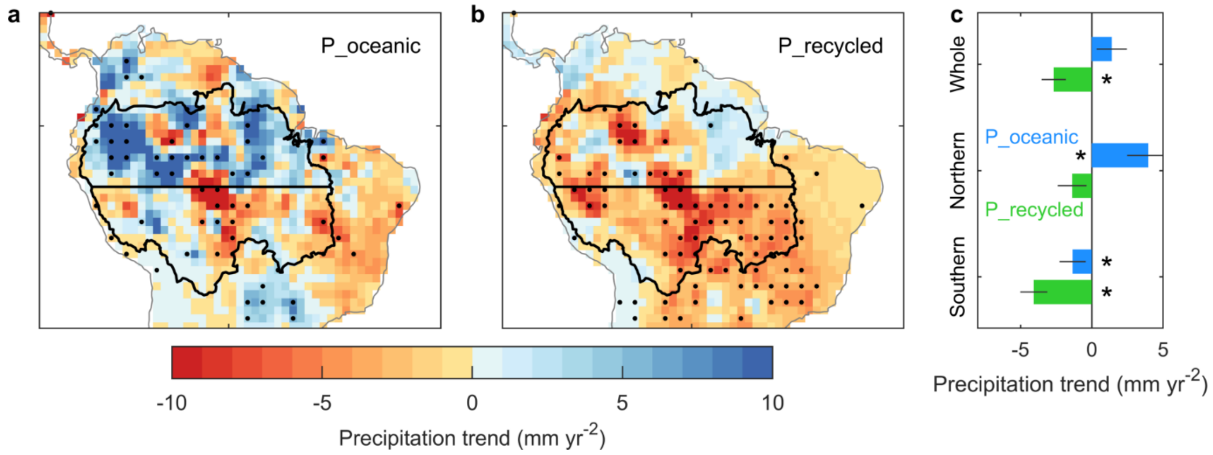

Supplementary Fig. 3. Observed precipitation trend and its moisture sources for the Amazon. The same as Fig. 1 but calculated for the period 1982-2016. **a,** Precipitation trend in the GPCP dataset. **b,** Precipitation trend in the GPCC dataset. The horizontal black line, at the latitude of 7.5°S, indicates our division between the northern and southern Amazon basins, while the outer black curve defines the full spatial extent of the Amazon basin. Stippling is for locations where the trend is statistically significant ($p < 0.05$). **c,** Precipitation trend averaged over the whole, northern and southern Amazon basins for the two precipitation datasets. Error bars represent the standard errors of the trends. Asterisks indicate that the trend is significant ($p < 0.05$). **d,** Direct oceanic contributions to precipitation trend (P_oceanic). **e,** Terrestrial recycled contributions to precipitation trend (P_recycled). **f,** Oceanic and terrestrial recycled contributions to precipitation trends averaged over the whole, northern and southern Amazon basins. In all panels, all trends are calculated for the period 1980-2019 inclusively. In **d-f**, P_oceanic and P_recycled are derived from atmospheric moisture tracking based on the GPCP dataset. Here P_total = P_recycled + P_oceanic.

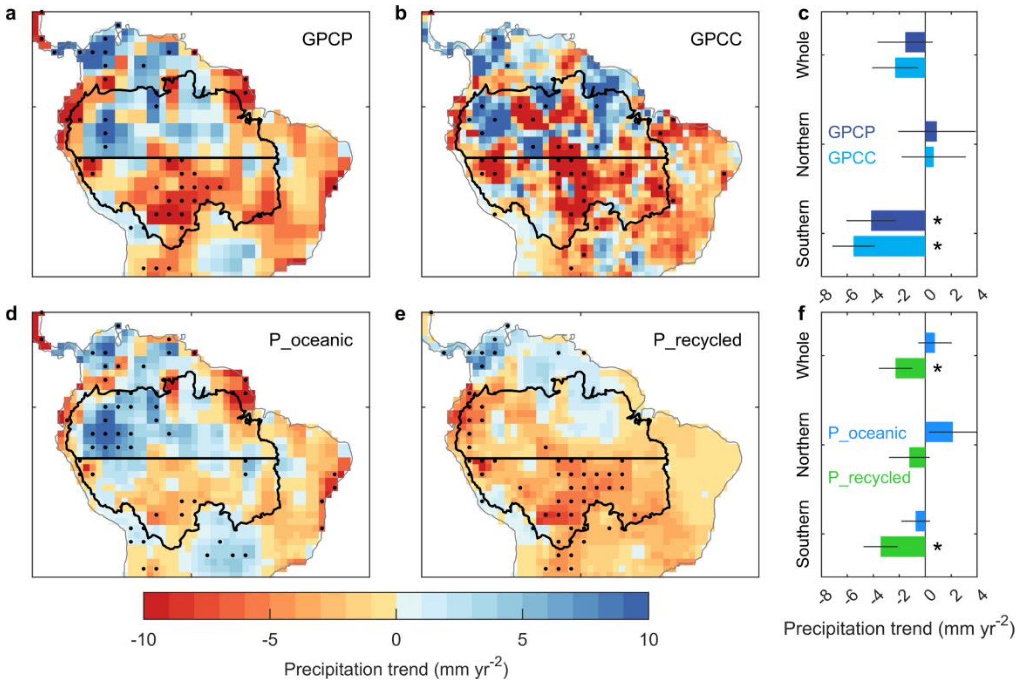

Supplementary Fig. 4. Climatological mean convective available potential energy (CAPE; a), distance of evapotranspiration (ET) transport (b) and ET fraction remained in local grid (c).

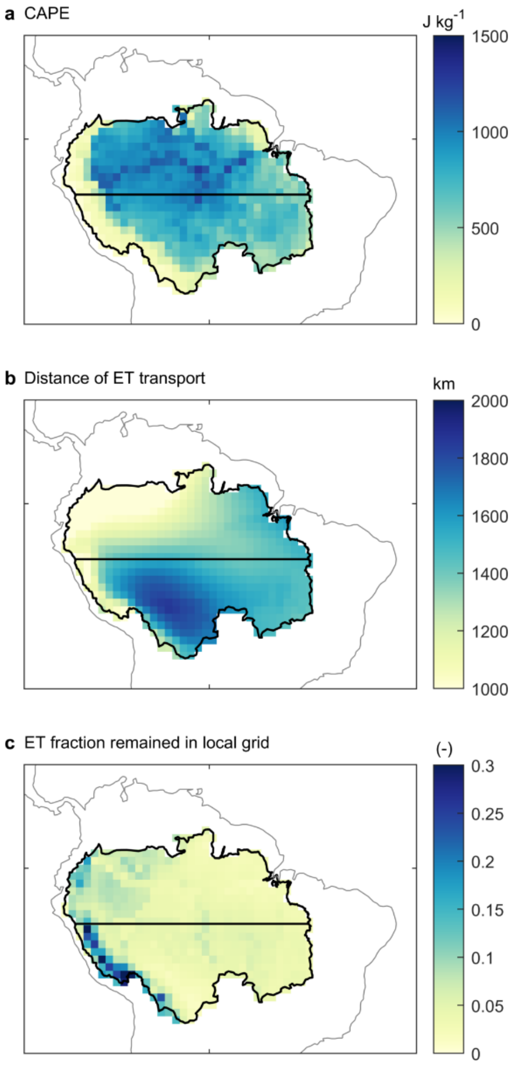

Supplementary Fig. 5. Projected changes in atmospheric circulation over the Amazon basin in the SSP2-4.5 scenario. a-b, Historical annual mean surface wind speed (**a**) and its change relative to the historical period (**b**) based on 29 CMIP6 climate models. The changes are calculated as the difference between the mean values in the last 20 years of 21st century (2081-2100) and that in historical baseline (1996-2015).

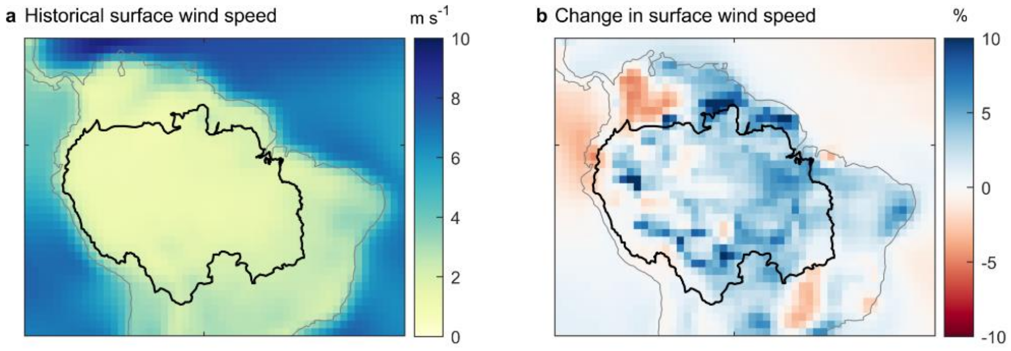

Supplementary Fig. 6. Projected change in recycled precipitation based a regional business-as-usual (BAU) deforestation scenario. a, The spatial pattern of forest cover change from the BAU scenario. **b,** Projected change in recycled precipitation. The blue and red lines mark the changes in weighted forest cover in the past 35 years and the BAU scenario, respectively, and the corresponding reductions in terrestrial recycled precipitation. The shaded areas represent the 95% confidence intervals of changes in the southern basin. The dashed black line indicates the extrapolation of historical relationship between changes in weighted forest and recycled precipitation.

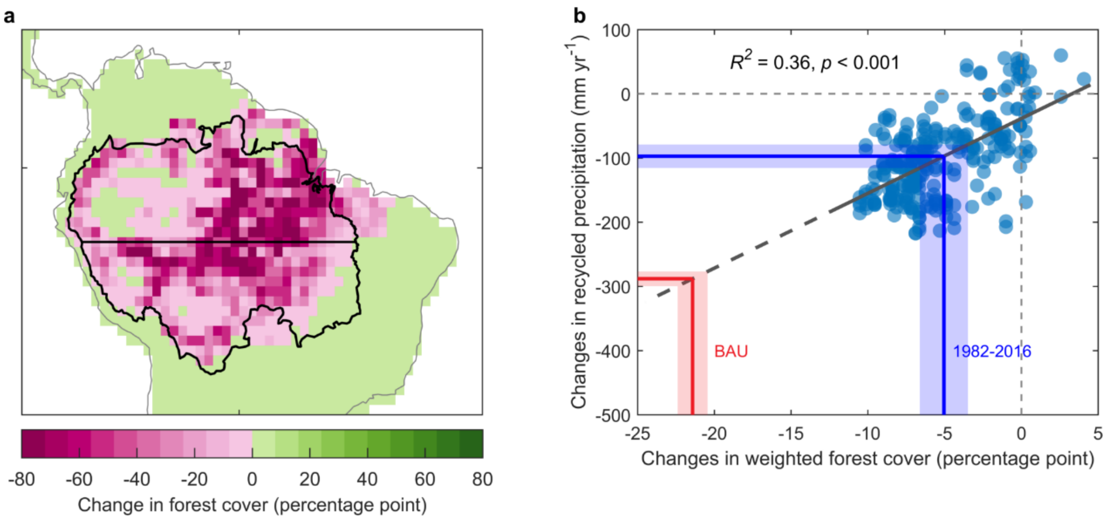

Supplementary Fig. 7. Precipitation trend averaged over the whole, northern and southern Amazon basins for the three observational periods. a, GPCP-based precipitation trend. **b**, GPCC-based precipitation trend. Error bars represent the standard errors of the mean trends.

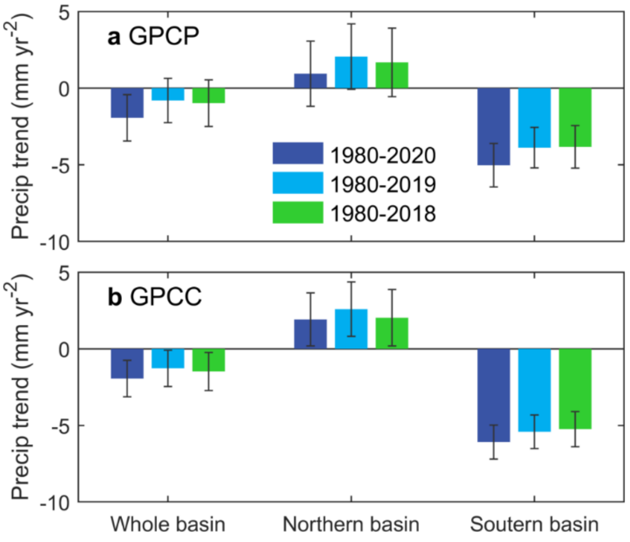

Supplementary Fig. 8. The ten sub-basins used to calculate water balance-based evapotranspiration within the Amazon basin. The sub-basins are distinguished by different background colours and numbered 1-10. Red points indicate the locations of hydrological stations (sub-basin outlets) and are labelled with the numbers used by the Global Runoff Data Centre (GRDC; Koblenz, Germany).

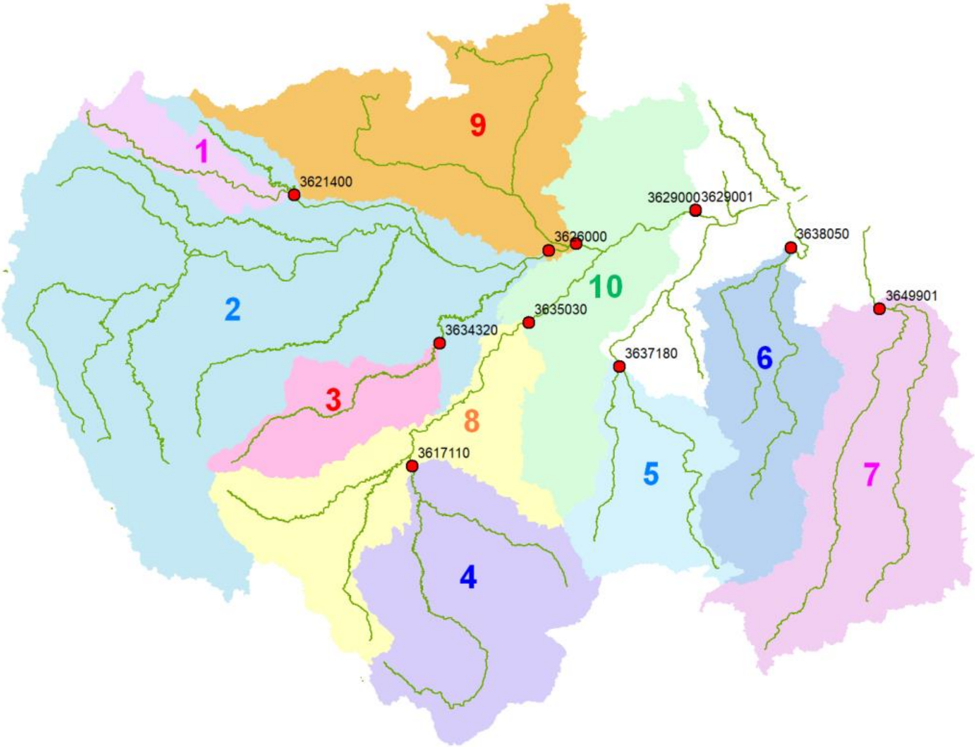

Supplementary Fig. 9. Comparison of water balance-based evapotranspiration (ET_wb) with GLEAM and FLUXCOM datasets over the Amazon basin. a-b, Climatological annual mean (**a**) and trend (**b**) in ET, averaged spatially over the Amazon basin. Error bars represent the standard errors of the means or trends. **c-h**, Spatial patterns of climatological annual mean (**c**, **e** and **g**) and trend (**d**, **f** and **h**) in ET for ET_wb, GLEAM and FLUXCOM estimates as marked. Stippling are locations where the trends are statistically significant ($p < 0.05$).

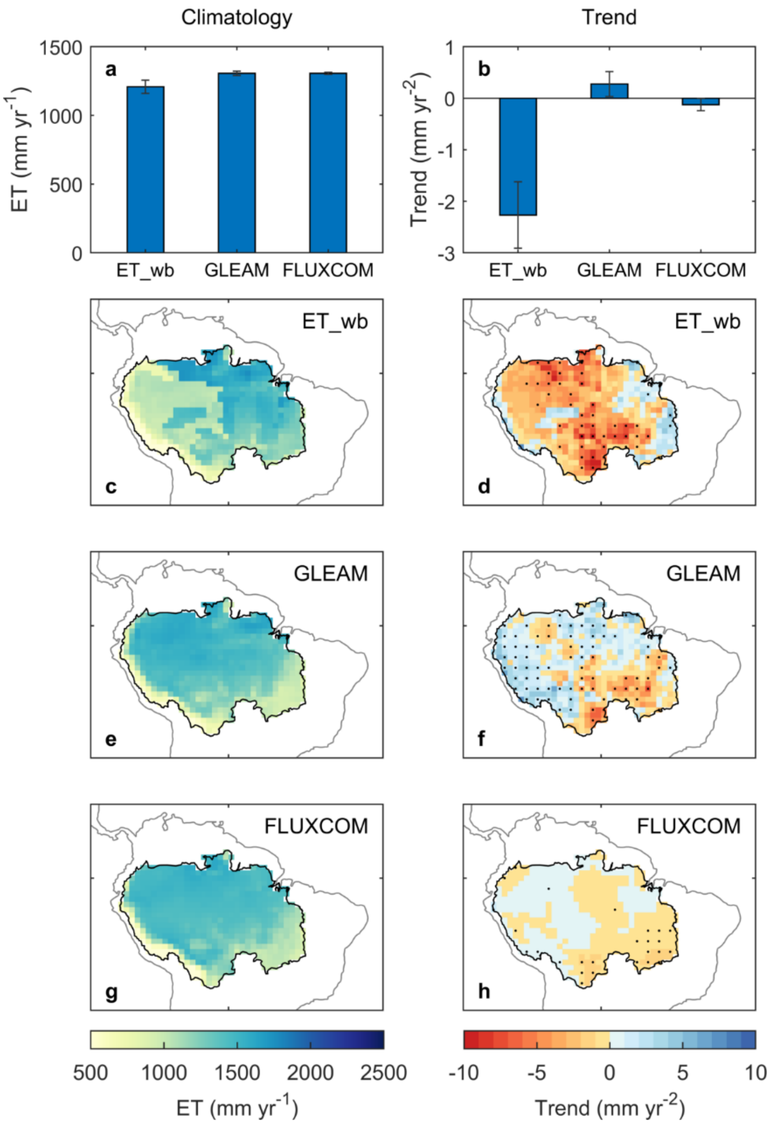

Supplementary Fig. 10. Comparison of water balance-based evapotranspiration (ET_wb), GLEAM and FLUXCOM against site-based flux observations. Error bars represent the standard errors of the means. The numbers on the bars of sites mean represent the average bias for the three ET products relative to site-based observations.

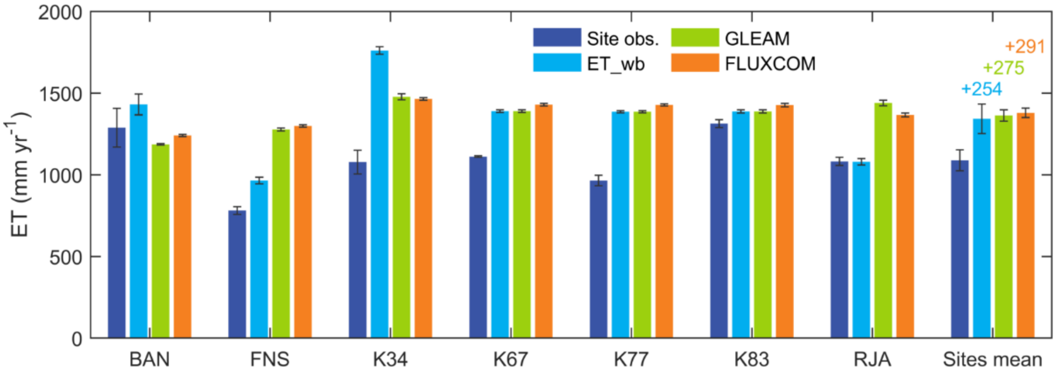

Supplementary Fig. 11. Projected changes in forest cover over the Amazon basin in SSP2-4.5 scenario. The forest cover change is calculated as the difference between the mean forest cover in the last 20 years of 21st century (2081-2100) and that in historical baseline (1996-2015). Primf and secdf represent primary and secondary forested lands, respectively.

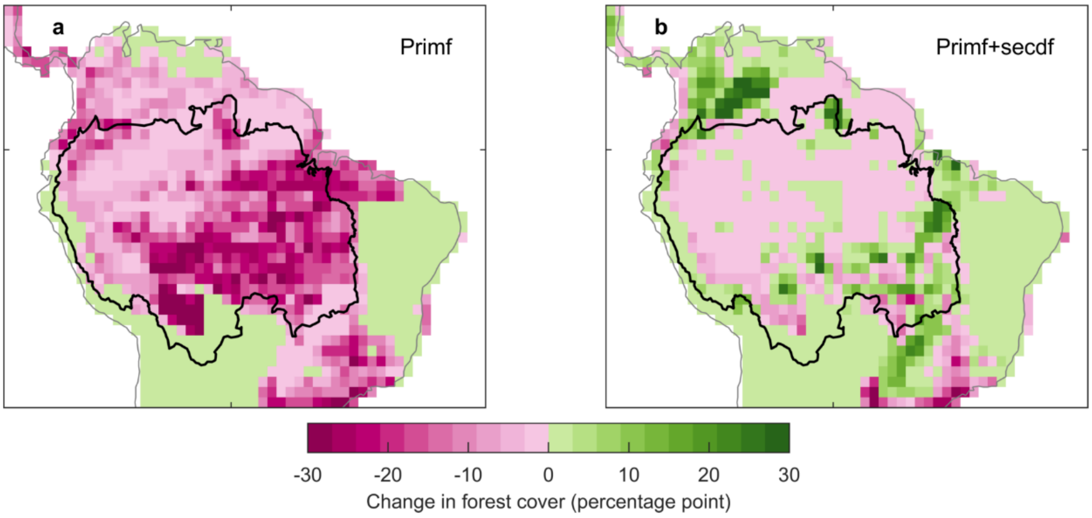

Supplementary Fig. 12. The impacts of forest cover change on recycled precipitation. Regression line (black line) is based on different spatial points, with each point representing local changes in terrestrial recycled precipitation and weighted forest cover in the southern Amazon basin for the period 1982-2016. Each point represents a 1° × 1° gridbox within the southern basin. Different from Fig. 4, recycled precipitation from WAM-2layers here is driven by evapotranspiration allowed to change everywhere (including the northern basin).

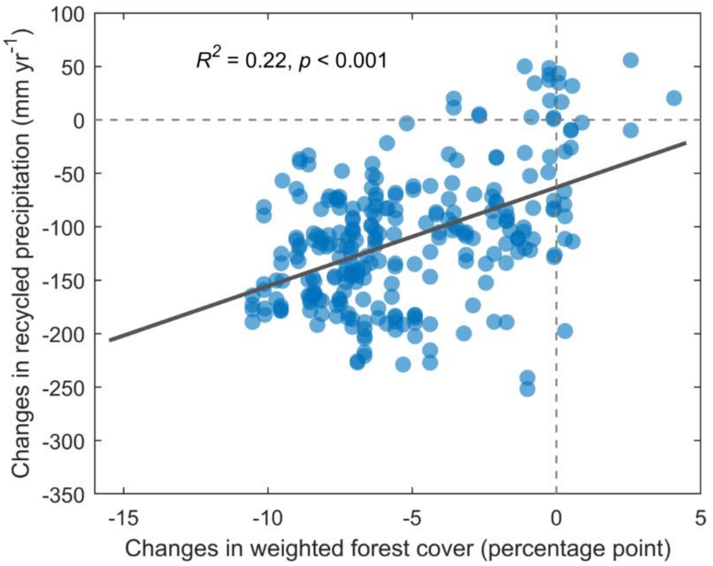

Supplementary Fig. 13. Evaluation of autocorrelation impact on the relationship between changes in weighted forest cover and recycled precipitation. Panel **a** shows the mean of forest cover data, which also evolves over time, at each location. Panel **b** is the spatial randomisation of the data in panel **a**, while preserving, for each moved point, the original timeseries. Panel **c** displays the trend in FC_w, derived from the randomised ('r') data, and so named rFC_w. Panel **d** is of identical format to Fig. 4a, except that the "*x*" variable is rFC_w. The blue arrows and labels indicate the data processing in each step between subplots.

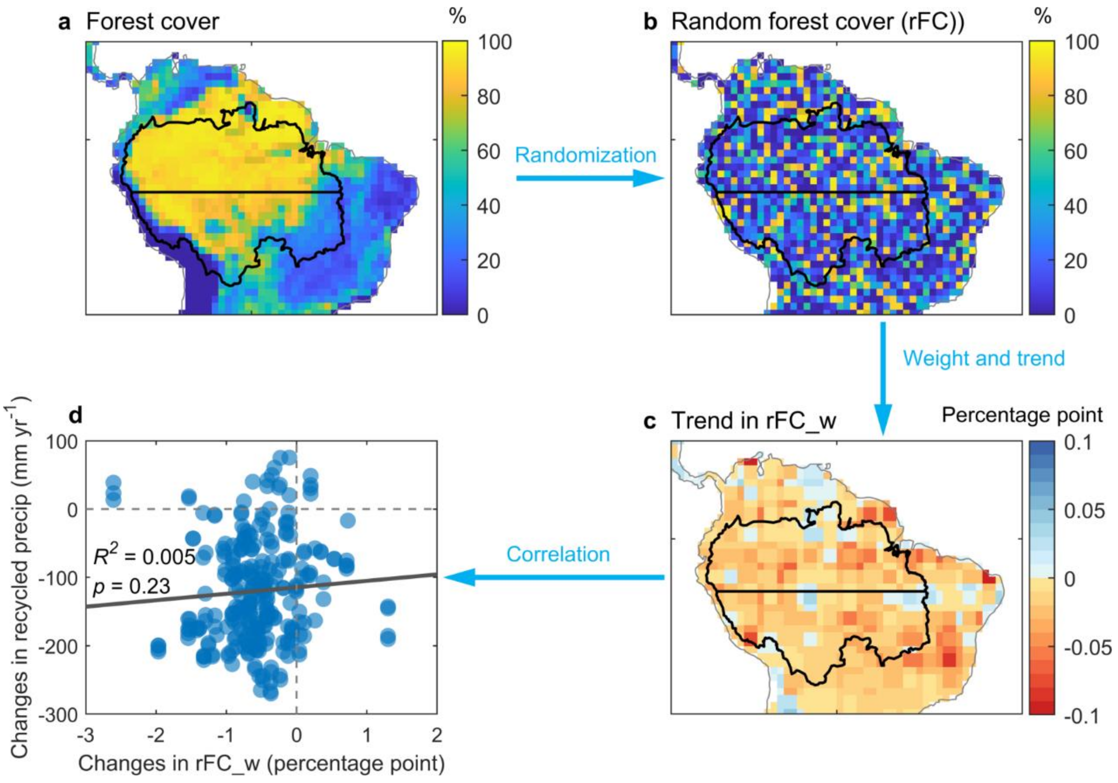

Supplementary Table 1. Dataset used in this study.

Dataset	Category	Resolution (degree)	Period	Reference
ERA5	Reanalysis	1.0	1979-2020	Hersbach, et al., 2020[1]
GPCP v2.3	Satellite	2.5	1979-2020	Huffman, et al., 2009[2]
GPCC v2022	Station-based	1.0	1979-2020	Schneider, et al., 2013[3]
GLEAM v3.5a	Satellite	0.25	1980-2020	Martens, et al., 2017[4]
FLUXCOM	Satellite + Station	0.5	1979-2016	Jung et al., 2019[5]
CMIP6	model	0.7-2.8	1996-2100	O'Neill, et al., 2016[6]
OAFlux	Satellite	1.0	1979-2020	Yu and Weller, 2007[7]
ISCCP_HXG	Satellite	0.1	1983-2018	Tang et al., 2019[8]
GRACE-REC	model	0.5	1979-2019	Humphrey and Gudmundsson. 2019[9]
VCF	Satellite	0.05	1982-2016	Song et al., 2018[10]
GRDC	Station	-	1979-2020	The GRDC[11]
LBA-ECO v2	Station	-	1999-2006	Restrepo-Coupe et al., 2021[12]
ET_fate	model	1.5	2001-2018	Link et al., 2020[13]

Supplementary Table 2. Characteristics and (dis)advantages of WAM-2layers.

Characteristics	Details
**Framework**	Eulerian approach (grid-based, 2D model)
**Vertical resolution**	Two-layer atmospheric model
**Key equations**	Atmospheric moisture budget (balance between evaporation, precipitation and moisture flux convergence at each time step)
**Input data**	1-hour precipitation and evapotranspiration (here constrained by observations); 6-hour specific humidity, zonal and meridional wind speeds at different pressure levels (from 1000 hPa to 100 hPa), and surface pressure from ERA5
**Application**	Tracks precipitation moisture sources and evapotranspiration sink (terrestrial vs. oceanic)
**Time step**	15 min
**Spatial resolution**	$1^{o} \times 1^{o}$
**(Dis)advantages**	**Details**
**Computational efficiency**	More efficient than Lagrangian models, suitable for large-scale and long-term simulations
**Vertical simplification**	Two-layer structure reduces complexity while capturing key vertical dynamics; it assumes well-mixed moisture within layers, but may suffer from imperfect vertical mixing especially in local-scale and short-term atmospheric processes
**Tracking precision**	Less effective than Lagrangian models in tracing specific moisture parcels over long distances or complex pathways

Supplementary Table 3. Comparison of water loss fraction with different data as input in WAM-2layers over the Amazon basin. P and ET represent precipitation and evapotranspiration, respectively.

	Input data		
	ERA-Interim	ERA5	ERA5 but with observational P and ET
Water loss fraction of tracked P	<0.1%	1.2%	1.8%
Water loss fraction of tracked ET	3.8%	4.6%	5.6%

Supplementary references

1 Hersbach, H. *et al.* The ERA5 global reanalysis. *Q. J. R. Meteorol. Soc.* **146**, 1999-2049, doi:https://doi.org/10.1002/qj.3803 (2020).

2 Huffman, G. J., Adler, R. F., Bolvin, D. T. & Gu, G. Improving the global precipitation record: GPCP Version 2.1. *Geophys. Res. Lett.* **36**, L17808, doi:https://doi.org/10.1029/2009GL040000 (2009).

3 Schneider, U. *et al.* GPCC's new land surface precipitation climatology based on quality-controlled in situ data and its role in quantifying the global water cycle. *Theor. Appl. Climatol.* **115**, 15-40, doi:10.1007/s00704-013-0860-x (2013).

4 Martens, B. *et al.* GLEAM v3: satellite-based land evaporation and root-zone soil moisture. *Geosci. Model Dev.* **10**, 1903-1925, doi:10.5194/gmd-10-1903-2017 (2017).

5 Jung, M. *et al.* The FLUXCOM ensemble of global land-atmosphere energy fluxes. *Sci Data* **6**, 74, doi:10.1038/s41597-019-0076-8 (2019).

6 O'Neill, B. C. *et al.* The Scenario Model Intercomparison Project (ScenarioMIP) for CMIP6. *Geosci. Model Dev.* **9**, 3461-3482, doi:10.5194/gmd-9-3461-2016 (2016).

7 Yu, L. & Weller, R. A. Objectively Analyzed Air–Sea Heat Fluxes for the Global Ice-Free Oceans (1981–2005). *Bull. Am. Meteorol. Soc.* **88**, 527-540, doi:10.1175/bams-88-4-527 (2007).

8 Tang, W., Yang, K., Qin, J., Li, X. & Niu, X. A 16-year dataset (2000–2015) of high-resolution (3h, 10km) global surface solar radiation. *Earth Syst. Sci. Data* **11**, 1905-1915, doi:10.5194/essd-11-1905-2019 (2019).

9 Humphrey, V. & Gudmundsson, L. GRACE-REC: a reconstruction of climate-driven water storage changes over the last century. *Earth Syst. Sci. Data* **11**, 1153-1170, doi:10.5194/essd-11-1153-2019 (2019).

10 Song, X. P. *et al.* Global land change from 1982 to 2016. *Nature* **560**, 639-643, doi:10.1038/s41586-018-0411-9 (2018).

11 GRDC, https://www.bafg.de/GRDC/EN/Home/homepage_node.html.

12 Restrepo-Coupe, N. *et al.* LBA-ECO CD-32 Flux Tower Network Data Compilation, Brazilian Amazon: 1999-2006, V2. *ORNL DAAC, Oak Ridge, Tennessee, USA*, doi:10.3334/ORNLDAAC/1842 (2021).

13 Link, A., van der Ent, R., Berger, M., Eisner, S. & Finkbeiner, M. The fate of land evaporation – a global dataset. *Earth Syst. Sci. Data* **12**, 1897-1912, doi:10.5194/essd-12-1897-2020 (2020).
